# Investigating Evolutionary Rate Variation in Bacteria

**DOI:** 10.1007/s00239-019-09912-5

**Published:** 2019-09-30

**Authors:** Beth Gibson, Adam Eyre-Walker

**Affiliations:** School of Life Sciences, University of Sussex, Brighton, BN1 9QG UK

**Keywords:** Bacteria, Evolutionary rate, Accumulation rate, Mutation

## Abstract

Rates of molecular evolution are known to vary between species and across all kingdoms of life. Here, we explore variation in the rate at which bacteria accumulate mutations (accumulation rates) in their natural environments over short periods of time. We have compiled estimates of the accumulation rate for over 34 species of bacteria, the majority of which are pathogens evolving either within an individual host or during outbreaks. Across species, we find that accumulation rates vary by over 3700-fold. We investigate whether accumulation rates are associated to a number potential correlates including genome size, GC content, measures of the natural selection and the time frame over which the accumulation rates were estimated. After controlling for phylogenetic non-independence, we find that the accumulation rate is not significantly correlated to any factor. Furthermore, contrary to previous results, we find that it is not impacted by the time frame of which the estimate was made. However, our study, with only 34 species, is likely to lack power to detect anything but large effects. We suggest that much of the rate variation may be explained by differences between species in the generation time in the wild.

**Electronic supplementary material:**

The online version of this article (10.1007/s00239-019-09912-5) contains supplementary material, which is available to authorized users.

## Introduction

Knowledge about the rates at which mutations arise and genomic change occur is crucial to understanding how organisms evolve and adapt and how molecular evolution proceeds. Evolutionary rates are known to vary extensively across species in both prokaryotes and eukaryotes and this variation will in part be associated with species characteristics and biology. Disentangling the factors that influence evolutionary rates have been explored in many animal and plant systems (e.g. (Bromham 2002; Smith and Donoghue 2008; Welch et al. 2008; Lanfear et al. 2010), but not so much in bacteria (though see Rocha et al. 2006; Weller and Wu 2015; Duchêne et al. 2016) Here, we investigate variation in the rate at which bacteria accumulate mutations through time in their natural environment over short time periods of a few months to a thousand years. We refer to these as accumulation rates to differentiate them from the mutation rate, the rate at which mutations occur, and the substitution rate, and the rate at which mutations fix in a species. These rates of accumulation are commonly estimated using temporarily sampled data (Drummond et al. 2003), or concurrent samples from a population with a known date of origin (e.g. from fossil dates or co-speciation events). They vary by orders of magnitude from species such as *Mycobacterium leprae* with an accumulation rate of 8.6 × 10^−9^ (Schuenemann et al. 2013) to species such as *Campylobacter jejuni* with a rate of 3.23 × 10^−5^ (Wilson et al. 2009).

It remains unclear why the rate at which mutations accumulate varies so much between bacteria. The accumulation rate *per year* must ultimately depend upon the rate of mutation *per year* and the probability that a mutation reaches sufficient frequency in the population to be sampled. If some mutations are caused by DNA replication, as seems likely in most organisms, then the mutation rate *per year* is a function of the mutation rate *per generation* and the generation time. The probability that a mutation reaches a certain frequency in the population depends upon natural selection, biased gene conversion and the effective population size. We consider each of these explanations in turn.

It has previously been shown that the time frame over which an accumulation rate is estimated can impact the estimate of evolutionary rate—they tend to be lower when measured over longer time frames (Ho and Larson 2006; Ho et al. 2011; Duchene et al. 2014; Biek et al. 2015; Duchêne et al. 2016). This effect is usually attributed to the inefficiency of purifying selection to remove slightly deleterious mutations over shorter time periods or problems with reliably estimating rates when the sequences are saturated. This pattern is evident in bacteria (Rocha et al. 2006; Biek et al. 2015; Duchêne et al. 2016), however, the evidence for the pattern is weak. In the most extensive analysis to date (Duchêne et al. 2016), the negative correlation between the accumulation rate and time frame was a consequence of just two species which had been sampled over a long time period. Furthermore, the authors removed datasets which showed no significant accumulation of mutations through time. This will have biased their analysis towards finding a negative correlation between the accumulation rate and sampling time frame, because species with slow accumulation rates will be removed from the analysis if they are sampled over short time frames, because they have not had enough time to accumulate significant numbers of substitutions.

Here, we revisit the question of whether the accumulation rate is slow in species sampled over longer time frames. We do this by comparing the rate of accumulation within species across different sampling times. We find little evidence for an association and consequently move on to explore other potential correlates of the accumulation rate. This includes (1) the mutation rate per generation (2) generation time and (3) the effectiveness of selection. We carry out analysis using both the raw values and also phylogenetic-independent contrasts (Felsenstein 1985) to account for phylogenetic-non-independence in the trait data.

## Materials and Methods

### Data Collection

We compiled estimates of the accumulation rates from the literature (Supplementary Table S1). For some species, we obtained multiple estimates and in most analyses we use the average of these (Supplementary Table S2). The genome size and GC content for each species is the average of all complete genomes on NCBI for each species. Nucleotide diversity estimates were calculated using orthologous sequence alignments for each species which were constructed using ODoSE ((Vos et al. 2013), https://www.odose.nl) and in-house scripts written in Python (https://www.python.org) (Supplementary Table S2). Lab-Doubling times were taken from (Vieira-Silva and Rocha 2010) (Supplementary Table S2).

All statistical analyses were performed in R (https://cran.r-project.org).

To estimate phylogenetic signal in the accumulation rates and all other traits we generated phylogenetic trees for the 34 species for which we have accumulation rate estimates (Supplementary Fig. S1). 16S rRNA sequences were downloaded from the NCBI genome database (https://www.ncbi.nlm.nih.gov/genome/) and aligned using MUSCLE (Edgar 2004) performed in Geneious version 10.0.9 (https://www.geneious.com, Kearse et al. 2012). From these alignments, maximum likelihood trees were constructed in RAxML (Stamatakis 2014) and integrated into the tests of (Pagel 1999) and (Blomberg et al. 2003) to the accumulation rates and all other traits implemented in the *phylosig* function in the R package *phytools v.0.6* (Revell 2012). Phylogenetic independent contrasts were carried out according to the method of Felsenstein (1985) using the *pic* function in *ape* v.4.1 (Paradis et al. 2004).

We averaged the accumulation rate estimates where we had multiple estimates from the same species. We recalculated the accumulation rates in two cases in which the number of accumulated mutations had been divided by an incorrect number of years: *E. coli* (Reeves et al. 2011) and *Helicobacter pylori* (Kennemann et al. 2011), see Gibson et al (2018) for details. We excluded some accumulation rate estimates for a variety of reasons. We only considered accumulation rates sampled over an historical timeframe of at most 1500 years. Most of our estimates of the accumulation rate are for all sites in the genome, so we excluded cases in which only the synonymous accumulation rate was given. We also excluded accumulation rates from hypermutable strains. Accumulation rate estimates used in the analysis are given in supplementary table S1.

The accumulation rate is expected to decrease as more divergent sequences are sampled because natural selection will remove deleterious genetic variation over time. To investigate this phenomenon quantitatively, we used a transition matrix to explicitly work out the distribution of allele frequencies *t* generations after a mutation was introduced into a haploid population. In the transition matrix, the first column represents the population when the mutation is first introduced. If there are *N* chromosomes in the population then there are *N* + 1 rows, where the first row represents loss of the mutation and the *N* + 1th row, fixation. The first column is therefore (0, 1, 0, 0, 0…0). To this column, we apply selection and drift. If the fitness of the wildtype is 1 and the fitness of the mutant is 1−*s* then the frequency after selection is $f^{\prime}(f,s) = {{(1 - s)f} \mathord{\left/ {\vphantom {{(1 - s)f} {(1 - sf)}}} \right. \kern-\nulldelimiterspace} {(1 - sf)}}$ where *f* the frequency before selection. To calculate the effects of drift we use the binomial distribution. Hence, the probability density of *x* copies of the mutation in generation *t* is

1 $$P\left( {N,x,s,t} \right) = \sum\limits_{i = 1}^{N - 1} {B\left( {N,x,i,s} \right)} P\left( {i,t - 1} \right)$$

where *B*(*N,x,i,s*) is the binomial distribution taking into account the effects of selection

2 $$B\left( {N,x,i,s} \right) = \frac{N!}{{x!\left( {N - x} \right)!}}\left( {f^{\prime}\left( {\frac{i}{N},s} \right)} \right)^{x} \left( {1 - f^{\prime}\left( {\frac{i}{N},s} \right)} \right)^{N - x}$$

By applying Eq. 1, we can work out the probability density of a mutation introduced in the first generation in subsequent generations; i.e. we calculate *P*(*x, *2) for all *x* from 0 to *N*, and then *P*(*x*, 3) for all *x* from 0 to *N*…etc.). The *i*th column and *jth* row represent the probability of observing a mutation introduced as a single copy at generation 1, in *j* copies in the *i*th generation. The chance that a sequence sampled in *t* generations in the future is different to the ancestral and can be calculated thus

3 $$D\left( {N,s,t} \right) = \sum\limits_{v = 1}^{t} {\sum\limits_{x = 1}^{N} {P\left( {N,x,s,v} \right)} } \frac{x}{N}$$

If we have two clones diverging from each other, then the overall divergence, assuming that mutations do not occur at the same site, which is reasonable for low levels of divergence, is twice this. We are interested in how selection affects the rate of accumulation and so we need to divide by the accumulation rate for neutral mutations, which is equivalent to dividing Eq. 3 by *t:*

4 $$A\left( {N,s,t} \right) = {{2D\left( {N,s,t} \right)} \mathord{\left/ {\vphantom {{2D\left( {N,s,t} \right)} {2D\left( {N,0,t} \right)}}} \right. \kern-\nulldelimiterspace} {2D\left( {N,0,t} \right)}}$$

In reality, not all deleterious mutations are subject to the same strength of selection so we sampled mutations from a gamma distribution; calculated *P*(*x,s,t*) for each and then averaged across mutations. We sampled 100 mutations for each set of parameters governing the distribution of fitness effects. *A *(*N,s,t*) is expected to scale in *N* generations, something we have confirmed; i.e. $A\left( {N,s,t} \right) = A\left( {zN,s,\frac{t}{z}} \right)$. We initially constructed a transition matrix with 100 chromosomes to study the pattern from 0 to 4* N* generations, but then subsequently investigated the pattern in more depth within the first 0.1* N* generations by constructing a transition matrix with 1000 chromosomes and the first 0.01* N* generations.

## Results

### Across Species

**Fig. 1:**
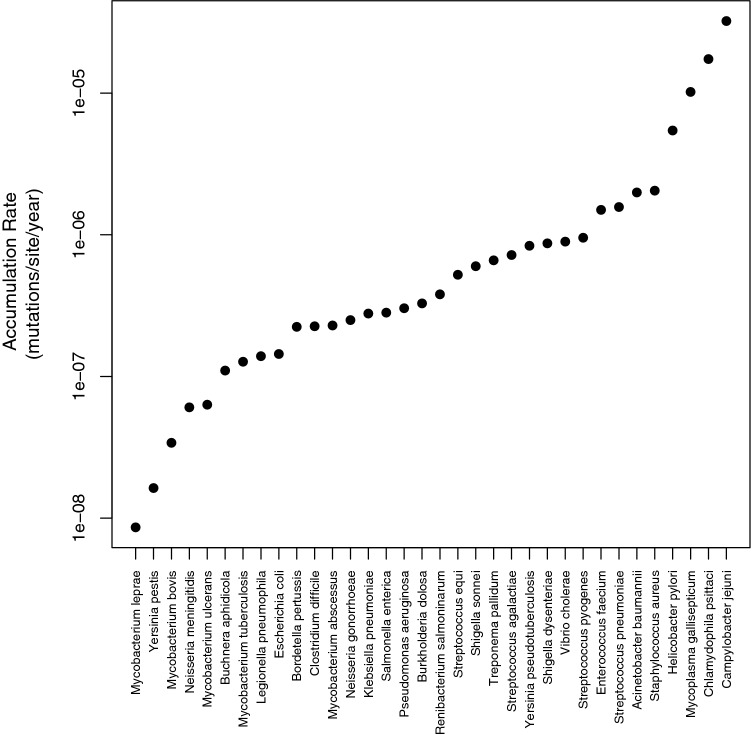
Distribution of accumulation rate estimates for 34 species of bacteria

We compiled estimates of the accumulation rate for 34 species of bacteria. These vary by over 3700 fold (Fig. 1.), but the majority of species accumulate mutations at rates of between 1 × 10^−6^ and 2 × 10^−6^ per site per year. In the sections below, we investigate what might cause this variation by looking for variables which correlate to the accumulation rate. Because the accumulation rate varies over orders of magnitude, all analyses were performed on the log of the accumulation rate. In such an analysis it can be important to correct for phylogenetic non-independence if there is a phylogenetic inertia. We have previously shown that the accumulation rate estimates show phylogenetic inertia (Gibson et al. 2018) (Table 1). To investigate the other variables we tested for phylogenetic inertia by inferring the phylogeny of our species using the 16S rRNA and then using the tests of Pagel (1999) and Blomberg et al. (2003). We find some of our other variables show phylogenetic inertia including genome size and GC content, but not all (Table 1).

**Table 1:** Tests of phylogenetic signal

Trait	λ	P value	K	P value
Accumulation rate	0.68	0.001	0.0005	0.37
Genome size	1	< 0.001	0.38	0.001
GC content	1	< 0.001	0.79	0.001
${{\pi_\text{N}} \mathord{\left/ {\vphantom {{\pi_\text{N}} {\pi_\text{S}}}} \right. \kern-\nulldelimiterspace} {\pi_\text{S}}}$	0.000062	0.99	0.0077	0.108
Lab DT	0.8	0.003	0.08	0.279

Pagel’s λ (Pagel 1999) and Blomberg et al.’s K (Blomberg et al. 2003)

### Sampling Time

The time-interval over which evolutionary rates are measured is thought to impact rate estimates so that they become slower when measured over longer time frames (Ho et al. 2011; Biek et al. 2015; Duchêne et al. 2016). This is as we might expect if a substantial fraction of mutations are mildly deleterious, since they would appear over a short time scale, but ultimately be removed by natural selection. Evidence for this effect comes from observation that the relative rate at which non-synonymous and synonymous mutations accumulate in bacterial genomes declines as a function of time (Rocha et al. 2006; Balbi and Feil 2007).

**Fig. 2:**
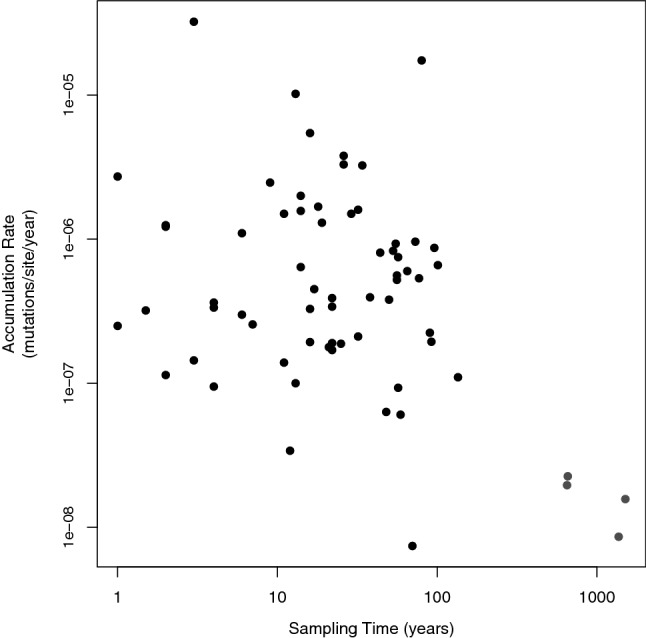
The accumulation rate vs sampling time. Yersinia pestis and Mycobacterium leprae are highlighted as outliers

**Fig. 3:**
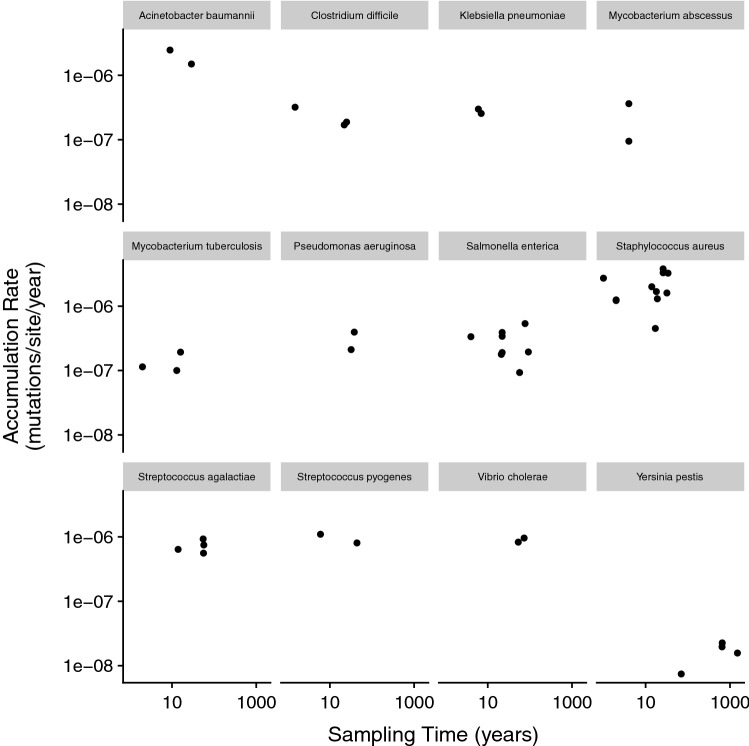
The accumulation rate vs sampling time split for the 12 species for which we have multiple estimates

We test whether the accumulation rate estimates scale negatively with sampling time, defined here as either the time-interval over which isolates were temporally sampled or the divergence time separating concurrent sequences. Sampling time varies from 1 year to just over 1500 years. We find a highly significant negative relationship between accumulation rate and sampling time (Fig. 2) (*r* = − 0.38, *p* = 0.0016) across all species for all studies, but this appears to be largely contributed by four points associated with two species, *Yersinia pestis* and *Mycobacterium leprae*. It is not clear whether *Y. pestis* and *M. leprae* have low rates because this is a feature of their evolution, irrespective of the time frame over which they were sampled, or because they have been sampled over long time frames. For several species, there are multiple estimates of the accumulation rate. If we control for any species effects by considering the correlation between the accumulation rate and the sampling time frame within these 12 species using ANCOVA, we find no correlation (slope = 0.022, *p* = 0.79) (Fig. 3). Furthermore, we find no relationship between the relative rates at which non-synonymous and synonymous mutations accumulate and the time frame over which the accumulation rate estimate was made (*r* = 0.2, *p* = 0.53), although for most datasets the accumulation rate was not calculated for the two types of site separately. In conclusion, we do not find strong evidence for a sampling time effect.

**Fig. 4:**
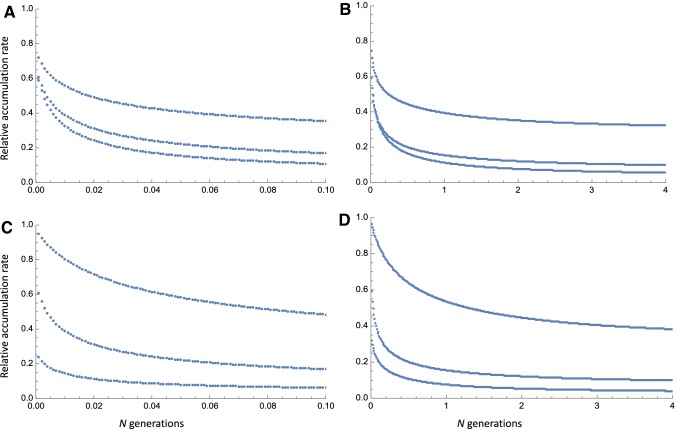
The expected relationship between the accumulation rate at selected sites relative to neutral sites and sampling time. In panels A and B, the shape parameter of the gamma distribution is varied 0.25 (top line), 0.50 (middle) and 0.75 (bottom); in panels C and D the mean strength of selection, multiplied by *N*, is varied from 10 (top), 100 (middle) and 1000 (bottom). Panels A and C show the relative accumulation rate over the first 0.1* N* generations, panels B and D over the first 4* N* generations

The absence of a relationship between the accumulation rate and sampling time might seem surprising given that there is ample evidence that slightly deleterious mutations segregate in bacterial populations; for example (Hughes 2005) showed that non-synonymous polymorphisms segregate at lower frequencies than synonymous polymorphisms in most species of bacteria. Thus, we would expect the rate of accumulation to decline as time progresses. To investigate this further, we derived the expected relationship between the accumulation rate and time using population genetic theory (see :"Materials and Methods"). We assume all mutations are drawn from a distribution of fitness effects (DFE), modeled as a gamma distribution, in which all mutations are either effectively neutral, or deleterious. We find, as expected, that the rate of accumulation declines. However, it is evident that it will be difficult to detect differences in accumulation rate unless accumulation rates are sampled over a very short time frame (< 0.1* N* generations, where *N* is the population size) and a much longer time frame (Fig. 4). This is because within a restricted time frame there is very little difference in accumulation rate.

### Mutation Rate

The rate at which bacteria accumulate mutations through time will in part be determined by the rate at which mutations occur per unit time. If some mutations are caused by DNA replication then the mutation rate *per year* will depend upon the mutation rate *per generation* and the generation time. We test each of these components in turn.

**Fig. 5:**
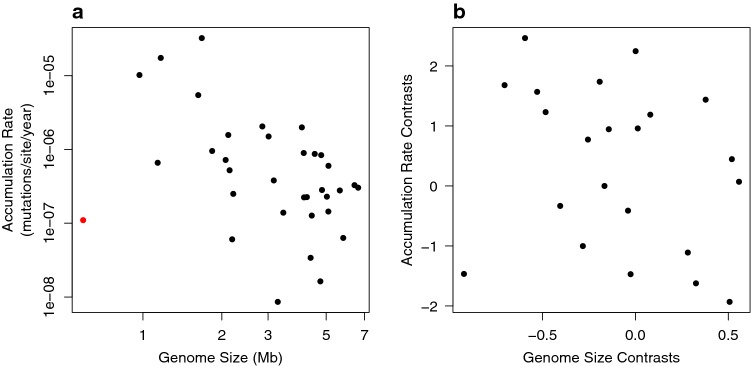
**a** The accumulation rate vs genome size (Buchnera aphidicola is highlighted as an outlier). **b** phylogenetic independent contrasts for the accumulation rate vs genome size

Unfortunately, it is difficult to directly test for a relationship between the accumulation rate and the mutation rate per generation because only five species in our dataset have estimates of both these rates. The correlation between the accumulation rate and mutation rate per generation is 0.07 (*p* = 0.9), but with such little information it’s difficult to determine whether a correlation exists. However, it is potentially possible to test the relationship between the accumulation rate and the mutation rate per generation indirectly because some genomic traits correlate to the mutation rate per generation. For instance, genome size is inversely correlated to the mutation rate/site/generation (Drake 1991; Lynch 2010; Lynch et al. 2016). We find a negative relationship between the mutation rate and genome size (*r* = − 0.68, *p* =  < 0.001), although this is mostly driven by *Mesoplasma florum* (Supplementary Fig. S2). The species used here are a different set of species to those used in the rest of the analysis and information on them can be found in supplementary table S3. A negative correlation between genome size and the accumulation rate has been previously observed for a range of viruses and bacteria (Lynch 2010; Biek et al. 2015) and we also find a strong negative correlation between the accumulation rate and genome size (Fig. 5a) (*r* = −0.43, *p* = 0.01) which becomes stronger when the obvious outlier *B. aphidicola* is excluded (*r* = −0.57, *p* =  < 0.001). The relationship is also negative, but loses significance, if we control for phylogeny using phylogenetic independent contrasts (PICs) after excluding low-variance comparisons and *B.aphidicola* (*r* = − 0.27, *p* = 0.23 (Fig. 5b). 10 comparisons were considered low variance as their standard deviations were < 0.21.

Genomic base composition may also correlate to the mutation rate per generation. GC content is known to vary greatly across bacterial species from less than 20% to over 70%. The origins of this variation remain unresolved. There is evidence that it is not solely a consequence of mutation bias (Hildebrand et al. 2010; Hershberg and Petrov 2010) and that biased gene conversion may be a factor (Lassalle et al. 2015). Given that the pattern of mutation is generally AT-biased in bacteria (Hershberg and Petrov 2010) (though see Long et al. 2015; Sun et al. 2017) variation in GC content due to selection or BGC can potentially generate variation in the mutation rate by shifting the GC content away from its equilibrium value (Krasovec et al. 2017). This effect may explain why *Mesoplasma florum*’s mutation rate is so high because although it has very low genomic GC content, the equilibrium GC content is predicted to be substantially lower (Krasovec et al. 2017). This will lead to positive correlation between the accumulation rate and GC content. The mutation rate may also be negatively correlated to GC content due to variation in effective population size; a low effective population size may lead to lower GC content but a higher mutation rate because selection on mutation rate modifiers is relaxed and repair genes are lost. Surprisingly we find a positive association between an inverse measure of *N*_*e*_($\pi$_N_/$\pi$_S_) and GC content (*r* = 0.473, *p* = 0.0094), although this is lost when we account for phylogenetic non-independence (*r* = 0.32, *p* = 0.168).

**Fig. 6:**
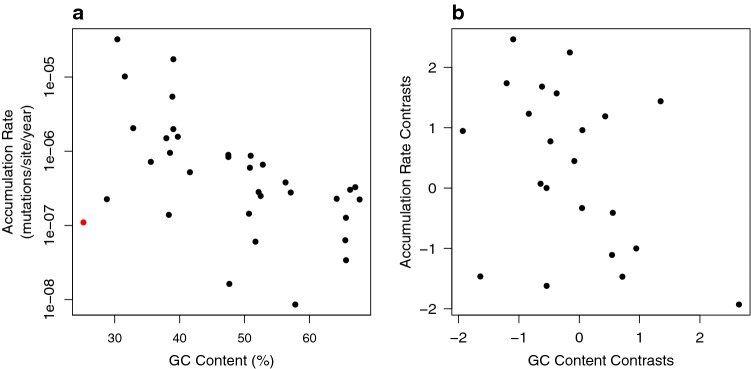
**a** The accumulation rate vs GC content (Buchnera aphidicola is highlighted as an outlier). **b** phylogenetic independent contrasts for the accumulation rate vs GC content

We observe a negative correlation between GC content and the mutation rate (*r* = − 0.59, *p* = 0.0016) (Supplementary Fig. S3), and we also find a strong negative correlation between the accumulation rate and the GC content (*r* = − 0.53 *p* = 0.001; Fig. 6a). Again, *B. aphidicola* is a conspicuous outlier and if removed the correlation is stronger (*r* = − 0.613, *p* =  < 0.001). This negative relationship is maintained and is almost significant for phylogenetic independent contrasts (− 0.390, *p* = 0.072) after exclusion of *B.aphidicola* and low-variance points (Fig. 6b).

We have detected moderately significant correlation between the accumulation rate and genome size and GC content. These two variables are correlated to each other but a multiple regression of accumulation rate versus both yields marginally significant results for GC content (*p* = 0.037) but not significant for genome size (*p* = 0.45) and neither come out significant when we control for phylogeny; it is, therefore, not possible for us to clearly resolve which might be the true correlate. Both could conceivably be linked to the mutation rate per generation. Under the drift-limit hypothesis, the mutation rate is expected to be negatively correlated to genome size, because larger genomes have potentially more deleterious mutations and this leads to more effective selection on the mutation rate (Lynch 2010; Lynch et al. 2016) GC content could be related to the mutation rate either through its correlation to genome size, a correlation for which there is no clear explanation, or because GC-content is a crude measure of how far a genome is from its equilibrium GC-content; if the mutation pattern is AT-biased then increasing GC-content increases the mutation rate (Krasovec 2017).

### Generation Time

It is likely that the accumulation rate should correlate negatively with generation time (or doubling time) because species with shorter generation times will accumulate more DNA replication errors per unit time. Eukaryotes appear to display this generation time effect (Bromham 2002; Smith and Donoghue 2008; Welch et al. 2008; Lanfear et al. 2010) and this is also evident in bacteria (Weller and Wu 2015) although see (Maughan 2007). Furthermore, the accumulation rate may also increase in populations that are rapidly expanding, for instance during epidemic disease, because of a reduction in generation time (Cui et al. 2013).

However, we find no relationship between the accumulation rate and the doubling time, as measured in the lab (*r* = -– 0.483, *p* = 0.0.60 for raw values and *r* = − 0.298, *p* = 0.21 for phylogenetic independent contrasts). Other genomic features also correlate to lab-doubling times (Vieira-Silva and Rocha 2010) but we find no correlation between the accumulation rate and 16 s gene copy number (*r* = 0.044, *p* = 0.802 for raw values and *r* = 0.126, *p* = 0.565 for phylogenetic independent contrasts) and tRNA abundance (*r* = − 0.085,*p* = 0.63 for raw values and *r* = 0.156, *p* = 0.47 for phylogenetic-independent contrasts. This may be because lab-doubling times do not reflect what occurs in the wild but they might relate to some aspect of bacteria life history. We have recently inferred the DT in the wild for 5 species of bacteria using an indirect method and we find no correlation between the wild DT and the DT in the lab (Gibson et al. 2018). Nevertheless, it is intriguing to note that some of the lowest accumulation rates come from species which are likely to have very long generation times in their natural environments. For example, *Mycobacterium leprae* grown on mouse footpads takes 300–600 h to replicate (Shepard 1960; Rees 1964; Levy 1976), and the endosymbiont *Buchnera aphidicola* takes between 175–290 h in its aphid host (Ochman et al. 1999; Clark et al. 1999). These two species have low-accumulation rates. *Mycobacterium tuberculosis*, which also has a low-accumulation rate, is thought to spend much of its time in a latent state during infection, which may cause it to have a low-accumulation rate (Colangeli et al. 2014). Other bacteria are known to form endospores which do not reproduce, including *Clostridium difficile*, which exhibits the lowest accumulation rate amongst the Firmicutes. Weller and Wu (2015) showed that the genomes of bacteria that contain a higher number of spore-forming genes tend to have slower evolutionary rates, suggesting that a generation time effect is present within these bacteria.

### Effectiveness of Selection

Selection and biased gene conversion will affect the probability that a mutation spreads to fixation in a population. Accumulation rates are estimated by excluding sites which are inferred to have been recombined and hence biased gene conversion is unlikely to explain the variation. In contrast, purifying selection will act to reduce the number of deleterious mutations surviving in populations, leading to a reduction the accumulation rate. How effective selection is at exerting its effects depends on the power of random genetic drift, i.e. the effective population size. We can potentially measure the effectiveness of selection by considering the ratio of the nucleotide diversity at non-synonymous and synonymous sites ($\pi$_N_/$\pi$_S_); populations with more efficient selection should have lower values of $\pi$_N_/$\pi$_S_. We consider the efficiency of selection using two sources of data; the ratio of the number of non-synonymous to synonymous polymorphisms, pN/pS, for the strains used to estimate the accumulation rate and $\pi$_N_/$\pi$_S_ in the species as a whole. We find no correlation between pN/pS in the strains to estimate the accumulation rate (*r* = 0.07, *p* = 0.84) but we have only nine data points. We find an almost significant correlation for the species wide $\pi$_N_/$\pi$_S_ and the accumulation rate (*r* = − 0.35, *p* = 0.062) but none if we control for phylogenetic inertia. (*r* = 0.1, *p* = 0.65).

### Lifestyle

We examined whether there are differences in the accumulation rate for bacteria with different lifestyles. Most of our species are pathogens and among these we divided them into obligate pathogens and opportunistic pathogens. We find that the accumulation rates do not differ significantly between these two groups (t test, *p* = 0.488). We further carried out an analysis controlling for phylogenetic non-independence by comparing sister pairs of species. We find no evidence that they are significantly different (paired sample t test, *p* = 0.947). Thus, lifestyle does not seem to have any clear impact on the accumulation rate.

### All Factors

We further carried out a multivariate analysis where we included all our variables into a multiple regression. When we consider the raw values, only genome size comes out as significant (*p* = 0.0153) and when we consider the phylogenetic independent contrasts lab-doubling times and $\pi$_N_/$\pi$_S_ come out as marginally significant with similar effect sizes (Standardized regression coefficient = 0.095, *p* = 0.080 and 1.01, *p* = 0.063 respectively); this suggests that accumulation rates may be higher in species with short lab DTs and smaller *N*_*e*_.

## Discussion

The rate at which bacteria accumulate mutations over short time frames of 1 to 1500 years varies by three orders of magnitude. The rate of accumulation must depend on the mutation rate per year and the strength of natural selection, and in turn the mutation rate per year is likely to depend on the mutation rate per generation and the generation time, assuming that at least some mutations are a consequence of replication errors. Potentially, variation in any of these factors—the mutation rate per generation, the generation time and the strength of selection—could be responsible for the variation in the accumulation rate.

Unfortunately, we find no very clear correlate of the accumulation rate; the accumulation rate is significantly correlated to the GC content and genome size, but neither factor is significant when we control for phylogeny. There is a hint that both lab DT and the effective population size may be important since these emerge as marginally significant in a multiple regression of all factors when we control for phylogeny. The lack of any clear correlate may be a result of the size of our dataset; we have data from just 34 species and many of the accumulation rates are estimated with considerable error. A simple power analysis suggests that if the true correlation was greater than 0.51 then 95% of the time we would reject the null hypothesis, so the correlation would need to be fairly strong. It is likely that the number of data points will increase considerably over the coming years and a more powerful analysis will be possible.

It has previously been shown that the accumulation rate is correlated to the timeframe over which the accumulation rate is measured (Duchêne et al. 2016). This relationship is expected given that deleterious mutations can segregate in a population, but these are ultimately removed from the population. However, in the study of Duchêne et al. (2016) the relationship was largely a consequence of two data points which were sampled over a very long time period, and Duchêne et al. excluded datasets in which there was significant increase in the accumulation of mutations with time. This would bias them towards finding a negative correlation between the accumulation rate and sampling time, because bacteria with slow accumulation rates would be excluded if they had been sampled over a short period of time because they would not show evidence of mutation accumulation. We found no evidence of a relationship between the rate of accumulation and sampling time within bacterial species suggesting that sampling time and accumulation rate are not correlated over the time frames being considered. This is perhaps not surprising because theoretical work suggests that differences in accumulation rate are only likely to be apparent if some bacteria are sampled over very short and very long time frames. The relationship is very likely to exist but we have been unable to detect it and it is clearly not responsible for most of the variation in the accumulation rate.

We find only very weak evidence that the accumulation rate is correlated to the doubling time, as measured in the lab. However, this is perhaps not surprising. Few bacteria probably double at anything like their lab measured rates in their natural environment. We have recently estimated the DT of five bacterial species indirectly by comparing their accumulation rate per year to their mutation rate in the lab per generation. Under the conservative assumption that the mutation rate per generation is the same in the wild and in the lab, one can estimate the number of generations per year by dividing the accumulation rate by the mutation rate. The five bacteria vary in their DT from a few hours to tens of hours. Conspicuously some of the DTs are much longer than their lab DT; for example, both *Escherichia coli* and *Salmonella enterica* can double every 30 min in the lab but Gibson et al. (2018) estimated their DTs to be 15 and 23 h, respectively. Gibson et al. (2018) also inferred the overall distribution of DTs in the wild across bacteria and estimated that DTs vary orders of magnitude from less than hour to 100 s of hours. This then would explain why accumulation rates vary so widely, there is a very large variance in DTs.

## Electronic supplementary material

Below is the link to the electronic supplementary material.

Supplementary file1 (PDF 55 kb)

Supplementary file2 (PDF 45 kb)

Supplementary file3 (PDF 46 kb)

Supplementary file4 (CSV 20 kb)

Supplementary file5 (CSV 2 kb)

Supplementary file6 (CSV 2 kb)
