# Supplementary material for: Investigating Evolutionary Rate Variation in Bacteria
Source: J Mol Evol. 2019 Sep 30;87(9):317–26. doi: 10.1007/s00239-019-09912-5 (PMC6858405; doi:10.1007/s00239-019-09912-5)
Supplement: Supplementary file 1 — Supplementary file1 (PDF 55 kb) [file 239_2019_9912_MOESM1_ESM.pdf]

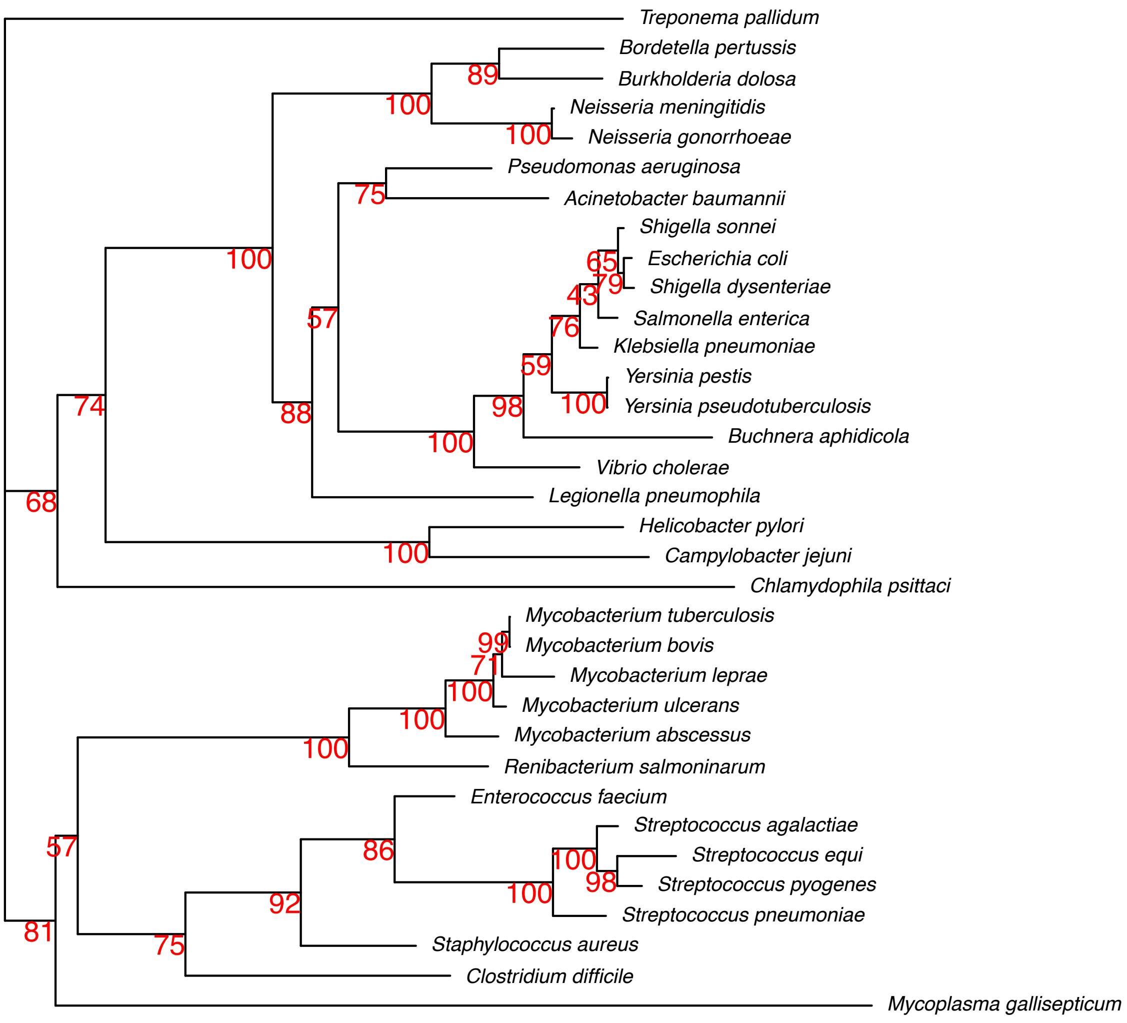

Supplementary Figure 1. 16s rRNA tree with bootstrap support values for the 34 species of bacteria for which we have an accumulation rate. This tree was used in phylogenetic analyses.
