# Supplementary material for: Investigating Evolutionary Rate Variation in Bacteria
Source: J Mol Evol. 2019 Sep 30;87(9):317–26. doi: 10.1007/s00239-019-09912-5 (PMC6858405; doi:10.1007/s00239-019-09912-5)
Supplement: Supplementary file 2 — Supplementary file2 (PDF 45 kb) [file 239_2019_9912_MOESM2_ESM.pdf]

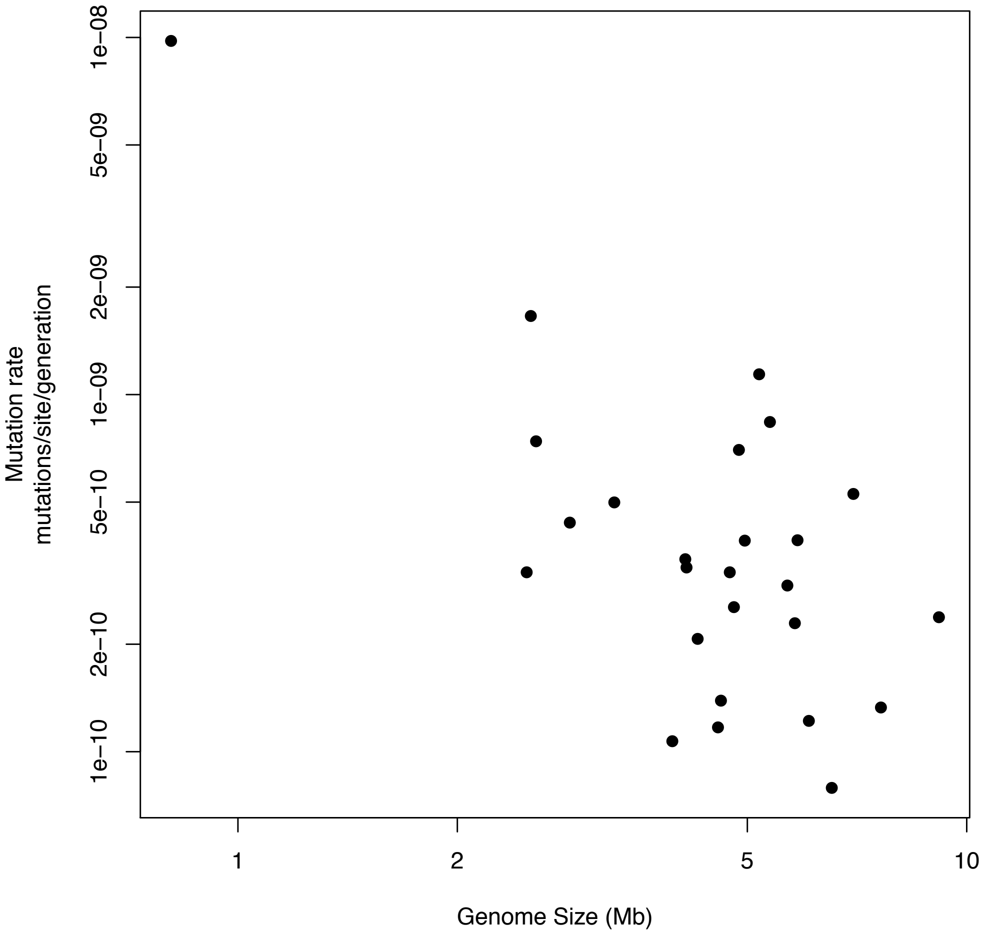

Supplementary Table S2. The mutation rate/site/generation vs genome size for 26 species of bacteria.
