# Supplementary figures and images for: Investigating Evolutionary Rate Variation in Bacteria
Source: J Mol Evol. 2019 Sep 30;87(9):317–26. doi: 10.1007/s00239-019-09912-5 (PMC6858405; doi:10.1007/s00239-019-09912-5)

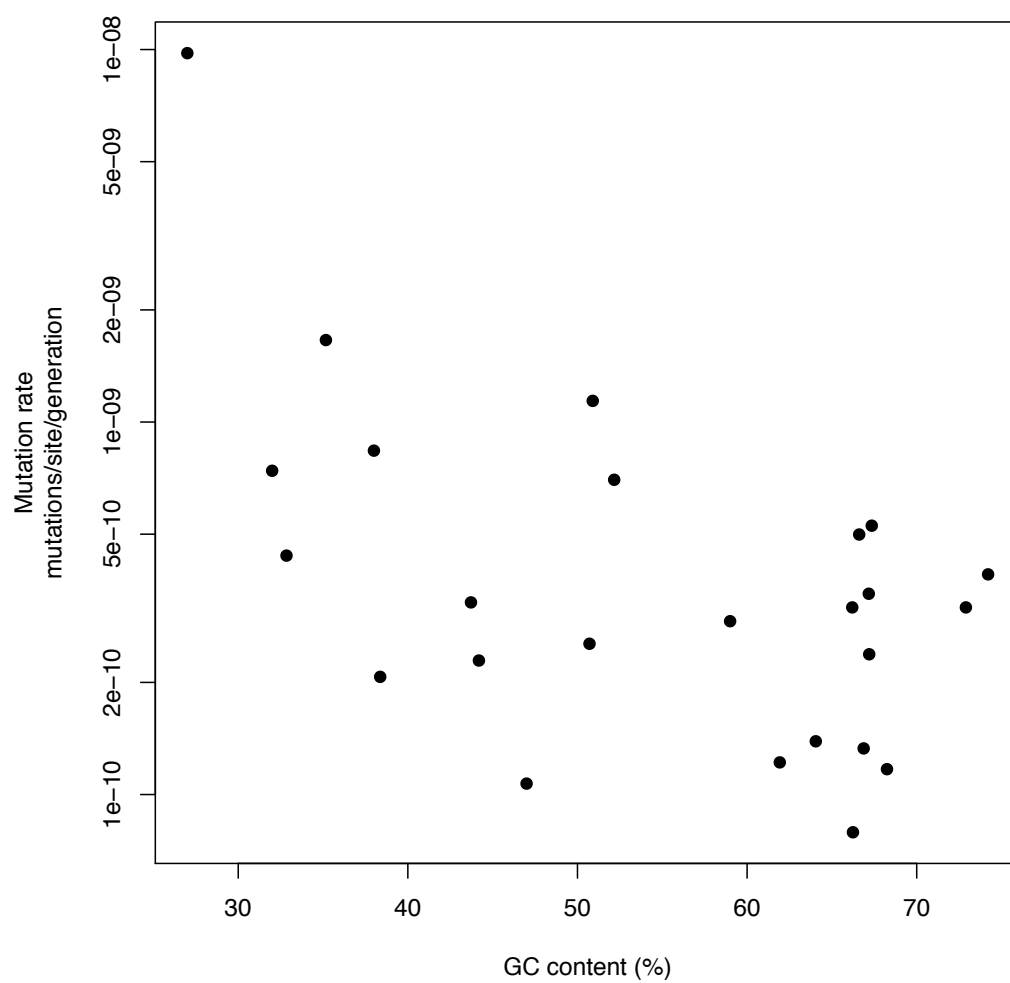

**Supplementary Fig. S3** The mutation rate/site/generation vs GC content for 26 species of bacteria.

Supplement: Supplementary file 3 — Supplementary file3 (PDF 46 kb) [file 239_2019_9912_MOESM3_ESM.pdf]
